# Proteomic analysis of HDL isolates reveals method-driven variability: An interlaboratory approach

**DOI:** 10.1016/j.jlr.2025.100957

**Published:** 2025-12-05

**Authors:** Francielle Aguiar Gomes, Douglas Ricardo Souza Junior, Michael Holzer, Graziella Eliza Ronsein

**Affiliations:** 1 Department of Biochemistry, Institute of Chemistry, University of São Paulo, São Paulo, Brazil; 2 Division of Pharmacology, Otto-Loewi Research Centre, Medical University of Graz, Graz, Austria; 3 BioTechMed Graz, Graz, Austria

**Keywords:** High-density lipoprotein, proteomics, ultracentrifugation, immunoaffinity, quantitative proteomics

## Abstract

The high-density lipoprotein (HDL) is the most heterogeneous and protein-rich lipoprotein, and its complex proteome has been correlated with many distinct properties. However, the lack of a standardized approach for HDL isolation, combined with the fact that different methods capture overlapping, but distinct HDL subspecies make the establishment of a composition-function relationship a challenge. Key factors influencing HDL proteomic profile are the isolation methodology and the technical variability associated with it. Importantly, interlaboratory technical variability associated with HDL isolation methodologies and how it affects the HDL proteome has never been determined. Here, we used two common methods to isolate HDL particles, ultracentrifugation (UC) and immunoaffinity chromatography against APOA1 (IAC), and performed a thorough evaluation of intralaboratory repeatability as well as intra- and interlaboratory reproducibility of these isolation methods, assessing their influence on HDL-associated proteins composition and abundance. Our results demonstrate that methodological variability has a greater impact on the HDL proteome than interlaboratory differences, with almost 60% of the variance in the data explained by the method of isolation. Importantly, the top 15 HDL proteins account for > 90% of the protein mass in HDL, regardless of the isolation method, and the variability in protein quantification is inversely associated with protein abundance. A joint analysis combining interlaboratory reproducibility of top 15 HDL proteins in multiple days shows 11 and 10 proteins with CV < 25% for UC and IAC, respectively. In summary, our findings demonstrate that standardized isolation methods can achieve acceptable reproducibility within and across laboratories, but they may capture distinct HDL particles.

High-density lipoprotein (HDL) encompasses a family of heterogeneous particles mostly known for the inverse association of their cholesterol levels with cardiovascular diseases (1), and their role in the reverse cholesterol transport (2). HDL particles have a complex and dynamic proteome, and studies have explored HDL proteome remodeling in a myriad of diseases states. However, reproducible results across multiple laboratories, as well as the establishment of a marker for such diseases, remain lacking. These unsatisfactory results can be attributed to four main factors: First, although close to 90% of HDL protein content is made up of apolipoprotein A1 (APOA1) and A2 (APOA2), the list of identified HDL proteins across different laboratories ranges from 13 to 487 proteins (3). Second, although HDL is formally defined by its density, multiple methods have been used to isolate its particles, and different approaches can enrich for overlapping but distinct HDL subspecies. Third, the reported number of proteins makes clear they are not present in the same particle; thus, the degree of subspeciation further increases the complexity. Fourth, without a consensus definition, many distinct methods of isolation have been used for HDL, but they may enrich for distinct subspecies and co-isolate a distinct set of plasma contaminants.

The most used strategy for HDL isolation is ultracentrifugation (UC), the method that was responsible for the definition of HDL as a unique class of lipoprotein particles decades ago (4). Given its high proportion of proteins to lipids, HDL floats at a density range of 1.063–1.21 g/ml, separating from plasma proteins and other lipoproteins (4, 5). Other methods based upon size, charge, and major lipoproteins content were later developed to obtain HDL particles from serum. Importantly, similar, but not identical particles can be isolated using distinct approaches. One example is the immunoaffinity chromatography (IAC), which is based on Alaupovic's apolipoprotein classification of lipoprotein particles (6). The use of specific antibodies for APOA1 coupled to a resin allows the separation of APOA1-containing particles from the entire size or density range presented (7).

In the past, methods based on different properties were indistinctly used to isolate HDL particles, but studies have shown that the method used to isolate HDL particles impacts their proteome composition and function (8, 9). Moreover, the technical variability associated with each HDL isolation methodology, assessed by experiments performed in independent laboratories have never been determined. Therefore, our study aims to systematically compare the impact on the HDL proteome of two different methods of HDL isolation, namely UC and IAC, performed in two independent research laboratories. For this purpose, we used the same serum pool and optimized protocols, and performed a thorough evaluation of repeatability as well as intra- and interlaboratory reproducibility of HDL isolation methods, assessing the influence of those methods on HDL proteome composition.

## Materials and methods

### Materials

Potassium bromide (KBr), ammonium bicarbonate, trifluoroacetic acid (TFA), Amicon Ultra-4 centrifugal filter and Vivaspin Turbo filter units, and Coomassie Brilliant Blue G-250 were purchased from Sigma-Aldrich. Sepharose 4 B coupled to a polyclonal anti-APOA1 antibody (#A81-104A) was purchased from Fortis Life Science. Dithiothreitol (DTT), iodoacetamide (IAA) and Bradford assay kit were purchased from Bio-Rad. Trypsin and ProteaseMax were purchased from Promega.

### Blood collection

Blood samples were collected from six apparently healthy donors, and serum was immediately obtained. All participants signed an informed consent form from the Institutional Review Board of the Medical University of Graz. Blood collection was approved by the local ethics committee (Nr.: 21–523 ex 09/10) and followed the principles of the Declaration of Helsinki. Baseline characteristics of the participants are available in Supplemental Table 1. A pool was prepared from the sera of the six subjects, frozen in multiple aliquots and used in all isolations performed by both laboratories.

### HDL isolation

#### Ultracentrifugation (UC)

HDL was isolated by density ultracentrifugation according to the literature with some modifications (9, 10). Serum density was adjusted to 1.24 g/ml (from an assumed background density of 1.006 g/ml) with KBr. For centrifugation, 13.5 ml Quick-Seal tubes (Laboratory 1 (Lab 1)) or 3.2 ml thick-wall polycarbonate tubes (Laboratory 2 (Lab 2)) were used (Beckman Coulter). To each tube, 10 ml (or 2.45 ml) of 1.063 g/ml KBr solution was added, and 3 ml (or 750 μl) of density-adjusted serum was carefully layered at the bottom. In Lab 1, the tubes were centrifuged at 88,500 rpm for 6 h at 4°C in a 90Ti fixed-angle rotor (Beckman Coulter), and in Lab 2, they were centrifuged at 110,000 rpm for 6 h at 5°C in a TLA-110 fixed-angle rotor (Beckman Coulter). Both protocols generated approximately 495,000 x *g* during centrifugation. HDL-containing fractions were desalted and buffer-exchanged using centrifugal filter units with a 3 kDa MWCO. In Lab 1, samples were concentrated with Vivaspin Turbo devices (maximum 4 ml) at 4,000 × *g* for 20 min and subsequently passed through a PD Midi column (Cytiva Life Science) equilibrated with PBS (1 ml load capacity). The eluate was further concentrated with two additional centrifugations using Vivaspin Turbo at 4,000 × *g* for 20 min each. In Lab 2, Amicon Ultra-4 devices (maximum 4 ml) were used for five consecutive centrifugations at 4,000 × *g* for 30 min, refilling with PBS after each spin. In both cases, the final wash was performed with 5% (w/v) sucrose for cryoprotection of HDL (11).

#### Immunoaffinity chromatography (IAC)

HDL was isolated as previously described (9). Briefly, 300 μl Sepharose 4B coupled to a polyclonal anti-APOA1 antibody was washed three times with PBS before the addition of 75 μl of serum, followed by an overnight incubation at 4°C. The unbounded fraction was washed away with PBS three times. Elution was carried out by incubating APOA1 resin with 3 M sodium thiocyanate for five minutes three times. The eluates were collected, combined and buffer exchanged with PBS using centrifugal filter units.

Each isolation method was performed independently in three days (three replicates per day) in two laboratories. The resulting HDL proteins were quantified using Bradford assay according to the manufacturer's instructions. Isolated HDL samples were evaluated by size exclusion chromatography and gel electrophoresis (under denaturing and native conditions).

### Gel electrophoresis

For reducing and denaturing gel electrophoresis (SDS-PAGE), isolated HDL (5 μg protein per lane) was separated on 12% polyacrylamide gels prepared in-house (Bio-Rad) using Tris-Glycine-SDS running buffer for 2 h at 100 V. For native gel electrophoresis, isolated HDL (5 μg protein per lane) was separated on a 6% polyacrylamide gel prepared in-house (Bio-Rad) using Tris-Glycine running buffer for 8 h at 50 V (at 4°C). Gels were run in a Mini-PROTEAN Tetra Cell (Bio-Rad) and stained with a freshly prepared solution of Coomassie Brilliant Blue G-250 as previously described (12).

### Size exclusion chromatography

An ӒKTA pure™ chromatography system (Cytiva Life Science) equipped with a Superdex 200 Increase 10/300 Gl column was operated with PBS (pH 7.4) as running buffer. HDL samples (50 μg) were loaded into the system using a 500 μl loop at a flow rate of 0.5 ml/min.

### Protein digestion

From each isolated HDL sample, 5 μg of protein were digested as previously described (13), with minor modifications. Proteins were diluted in 50 mM ammonium bicarbonate buffer containing 0.01% ProteaseMax, reduced with 5 mM DTT for 1 h (at 37°C) and alkylated with 15 mM IAA for 30 min (at room temperature, protected from light). The excess IAA was quenched using 2.5 mM DTT for 15 min at room temperature. Proteins were digested with freshly prepared trypsin at a ratio of 1:40 (trypsin:proteins, w:w). After 4 h, a second trypsin aliquot was added to the samples (1:50, trypsin:proteins) followed by an overnight incubation at 37°C. Digestion was stopped with 0.5% TFA. Samples were desalted using the C18-StageTip protocol (14), dried under vacuum and stored at −80°C until further analysis. Prior to MS analysis, samples were resuspended in 0.1% formic acid.

### LC-MS/MS analysis

All data were acquired in a Nano EASY-nLC 1200 coupled to an Orbitrap Fusion Lumos mass spectrometer, using a nanospray Flex NG ion source (Thermo Fisher Scientific). Digested HDL proteins (100 ng) were loaded onto a NanoViper trap column (C18, 3 μm, 75 μm × 20 mm, Thermo Scientific) and washed with 20 μl of 0.1% formic acid (solvent A) at a maximum pressure of 500 bar. Trapped peptides were eluted onto an Acclaim PepMap analytical column (C18, 2 μm, 75 μm × 150 mm, Thermo Scientific) at a flow rate of 300 nl/min. The chromatographic gradient was 5%–28% solvent B (80% acetonitrile with 0.1% formic acid) for 25 min; 28%–40% solvent B for 3 min; and 40%–95% solvent B in 1 min. Then, the column was washed for 10 min with 95% solvent B (350 nl/min) before re-equilibration of the system with solvent A for the next injection. The mass spectrometer was operated in positive ion mode using Orbitrap as the mass analyzer. Internal calibration with polydimethylcyclosiloxane ion (*m/z* = 445.12003) was performed during all analyses. Samples were acquired in data-independent acquisition (DIA) mode, with monitored predefined staggered precursor isolation windows of 24 *m/z* (15). Orbitrap resolution was set to 30,000 (at *m/z* 200), injection time of 54 ms and precursor scan range from 400 to 1000 *m/z*. Precursor ions were fragmented by HCD with a normalized collision energy of 30, in centroid mode and AGC target of 5 × 10^5^.

### Data processing

Thermo Scientific raw files obtained from DIA runs were converted to mzML files using MSconvert (v.3.0.11781). The converted files were upload in DIA-NN (v.1.9.2.0) (16) and searched against Homo sapiens Uniprot database (download on July 2024; 20,650 entries using a library-free approach. Default DIA-NN settings were used. They were as follows: Mass accuracy and MS1 accuracy, 0; Scoring, generic; Proteotypicity, genes; Machine learning, NNs (cross-validated); Quantification strategy, QuantUMS (high precision); Cross-run normalization, RT-dependent; Library generation: IDs, RT & IM profiling. The options match-between-runs (MBR) and protein inference were enabled. Trypsin was defined as the protease with two missed cleavages allowed. Cysteine carbamidomethylation and *N*-terminal methionine excision were selected as fixed modifications and oxidized methionine as variable modification. A protein was considered identified by the isolation method if it was detected in at least two out of three replicates on two separate, non-consecutive isolation days. In other words, the protein had to be present in at least two replicates on one day and in at least two replicates on another non-consecutive day. DIA-NN's normalized intensity values were used to calculate protein relative abundances within each sample. For the repeatability and reproducibility studies, all peptide-level quantitative results were exported from DIA-NN and imported into Skyline (16) for peak integration verification and manual inspection of all transitions to ensure correct peak detection. For each protein, 2–16 peptides were selected (Supplemental Table 2), and the protein intensity was determined by summing the peptide intensities and normalizing by the total ion chromatogram (TIC). These validated Skyline results are available in Supplemental Table 3 and were used for subsequent statistical analyses.

Protein intensities were log_2_-transformed for Bland-Altman analysis and multiple-sample test with a permutation-based false discovery rate (FDR) of 5% followed by Z-score normalization for heatmap construction. Only proteins commonly identified in UC and IAC methods were included in the principal component analysis (PCA). All integrated peaks were manually inspected using Skyline software (17) to ensure correct peak detection and integration. Figures were generated in R (v. 4.1.2) using the R packages “ggplot”, “VennDiagram”, and “pheatmap”. Protein symbols were derived from gene symbols as per the recommendations of the Human Genome Organization Gene Nomenclature Committee, and were written in all capital letters and not italicized.

## Results

### Experimental workflow

**Fig. 1:**
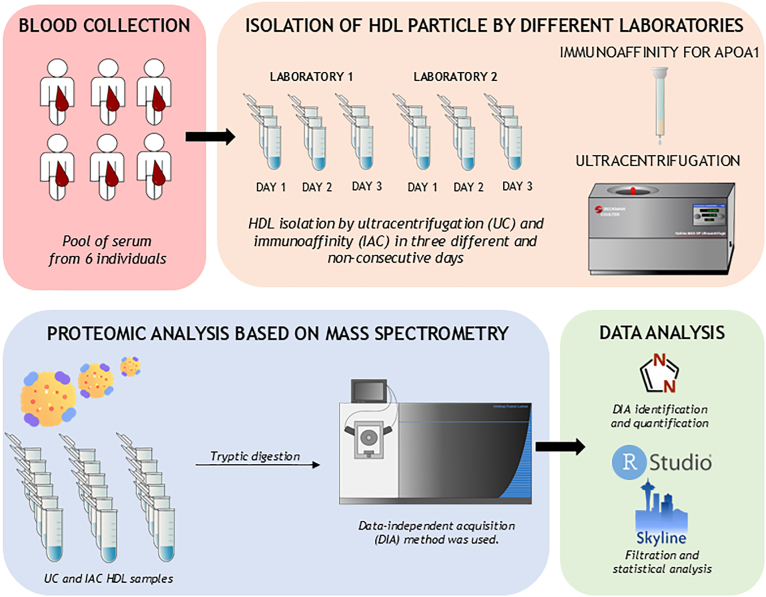
Workflow for interlaboratory comparison of HDL isolation methods by proteomics. Schematic representation of the experimental design of the study. Samples were obtained from a serum pool of six apparently healthy donors and used by both laboratories to isolate HDL particles by UC and IAC. For each laboratory, isolations were carried out in triplicate in three non-consecutive days. DIA was used as the acquisition method, protein quantification and statistical analyses using DIA-NN, Skyline and RStudio software.

This study aimed to systematically evaluate the interlaboratory reproducibility of two common techniques used to isolate HDL particles, focusing on the consistency of their associated proteome. Briefly, the same serum pool obtained from six apparently healthy individuals was processed in two independent laboratories across two continents to obtain HDL particles by UC and IAC against APOA1. The baseline characteristics of the subjects are available in Supplemental Table 1. The laboratories, randomly named Laboratory 1 (Lab 1) and Laboratory 2 (Lab 2), used equivalent protocols, and performed HDL isolation for each methodology in triplicate and in three independent days. To minimize other sources of variation, sample processing for LC-MS/MS analysis was performed simultaneously in the same laboratory, and it was followed by the randomized injection of samples using a previously standardized DIA proteomic methodology (15, 18). A summary workflow is provided in Fig. 1.

### Size-exclusion chromatography reveals a distinct distribution of HDL particle sizes for UC and IAC

**Fig. 2:**
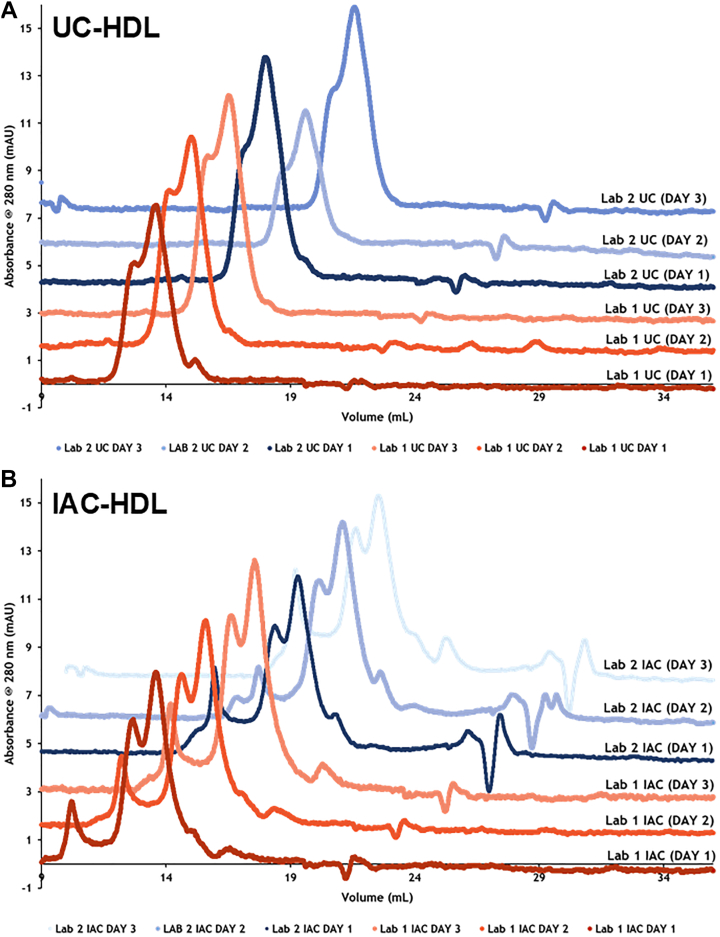
Size-exclusion chromatography of isolated HDL samples. Isolation of UC-HDL (A) and IAC-HDL (B) by both laboratories performed as described in the methodology section. Orange lines indicate laboratory 1 (Lab 1) and blue lines indicate laboratory 2 (Lab 2). The individual elution profiles were uniformly shifted for visualization purposes, but not modified in terms of scale or peak shape.

Size-exclusion chromatography was performed to evaluate the profile of HDL particles obtained by UC and IAC methods on different days (Fig. 2). The results indicate similar profiles for all HDL samples, regardless of isolation methodology or laboratory. However, IAC-isolated HDL particles present a small peak eluting in approximately 9 ml that is absent in UC-isolated HDL samples. This peak was previously reported (9). The HDL profile was also evaluated by SDS-PAGE and native gel electrophoresis (Supplemental Fig. S1), showing a similar pattern for HDL-associated proteins.

### DIA proteomics shows UC- and IAC-isolated HDL particles have a distinct proteome

**Fig. 3:**
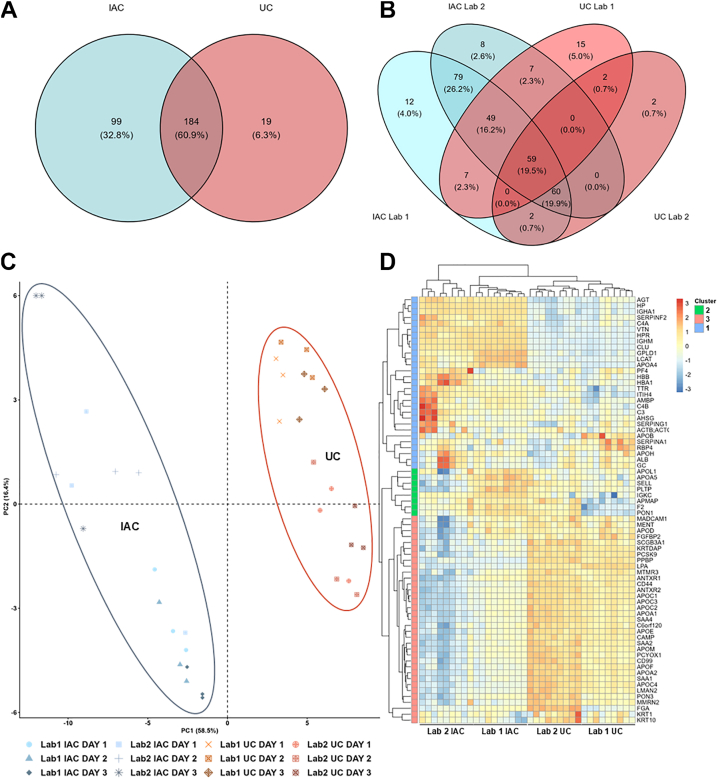
The protein composition of HDL particles varies according to the isolation method (UC or IAC). A-B: Venn diagrams showing unique and shared proteins between different isolation methods. Laboratories are clustered together (A) or independently evaluated (B). C: Principal component analysis (PCA) plot based on log_2_-transformed and protein intensities based on DIA-NN quantification. Each point represents one replicate, colored by isolation method (UC or IAC) and shaped by laboratory. PC1 explains 58.5% of the total variance and clearly separates samples by isolation method, whereas PC2 accounts for intra-method variability across laboratories. D: Heatmap generated from DIA proteomics data (log_2_-transformed intensities) for proteins consistently detected in all replicates. Rows represent proteins, and columns represent individual replicates (grouped by method and laboratory). Color scale reflects relative abundance (Z-score per row).

To be considered as pertaining to HDL particles isolated by a given method, the protein had to be identified in at least two out of three replicates in two out of three isolation days. Taken isolations carried out by Lab 1 and Lab 2 together, 302 proteins were identified by mass spectrometry-based proteomics, with 184 (60.9%) of them shared by both techniques (Fig. 3A), but only 59 proteins (19.5%) were found in HDL samples isolated by both laboratories and techniques (Fig. 3B). Nineteen proteins were found exclusively in HDL samples isolated by UC, but only two of them were shared by both laboratories (keratin 5 – KRT5, a contaminant protein from sample preparation –, and Cystatin C – CST3). Fifteen proteins were found only in samples isolated by UC in Lab 1, examples of these proteins include Protein S100A7 (S100A7), Peroxiredoxin-2 (PRDX2), and Pigment Epithelium-derived Factor (SERPINF1). Ninety-nine proteins were exclusively identified in HDL samples isolated by IAC methodology, almost 80% of them (n = 79) were commonly detected in samples isolated by Lab 1 and Lab 2. Many of these proteins (41/99) belong to immunoglobulin chains, protease inhibitors (SERPINA4 and SERPINA10), and complement system, such as complement factor B and I (CFB and CFI) and complement components 5, 6 and 7 (C5, C6 and C7). Interestingly, cholesteryl ester transfer protein (CETP) was consistently detected only in HDL samples isolated by IAC; thus, studies aiming to quantify CETP protein in HDL should take this into consideration. Twelve proteins were detected only in samples isolated by IAC by Lab 1, while eight proteins were detected only in IAC samples isolated by Lab 2. Isolation of HDL by IAC may isolate APOB-containing lipoproteins that also carry APOA1, but in this study, APOB was also detected in samples isolated by UC, with minimal differences in abundance between techniques. The complete list of proteins identified in UC and IAC isolation methodologies is available in Supplemental Tables 4 (overall) and 5 (for each isolation day).

Principal component analysis (PCA) of HDL proteome shows that the majority of the variance among samples is explained by the isolation method, with a clear distinction between UC-HDL and IAC-HDL, according to the first principal component (PC1, 58.5% of total variability) (Fig. 3C). The second principal component (PC2, 16.4% of total variability) explains the much less pronounced variance in HDL proteome composition caused by two distinct sources, the laboratory that samples were isolated, and the isolation day. The heatmap in Fig. 3D confirms the results illustrated by the PCA, showing samples clustered first by isolation method and then by isolation laboratory (columns). After filtering out exclusive proteins for each specific methodology, the differentially abundant proteins were grouped in three main clusters (heatmap rows, multiple-sample test with a permutation-based FDR of 5%). Cluster 1 is composed of proteins with higher abundance in IAC-HDL, cluster 2 shows proteins with discordant isolation results (proteins more abundant in IAC-HDL isolated by Lab 1 and in UC-HDL isolated by Lab 2), while Cluster 3 encompasses proteins more abundant in UC-isolated HDL. IAC-isolated HDL (Cluster 1) is enriched in clusterin (CLU), immunoglobulins (IGHM and IGHA), complement proteins, and apolipoprotein A4 (APOA4). The higher abundance of these proteins is likely related to the presence of a subclass of large HDL particles, overrepresented in IAC isolation methodology, as seen in Fig. 2, and previously reported by us (9). Moreover, protein isolation using Sepharose as a matrix might enrich for complement proteins (19). On the other hand, UC-isolated HDL (cluster 3) shows enrichment for the majority of the apolipoproteins, such as APOM, APOC1, APOC2, APOC3, and APOA1—the most abundant protein of HDL particles. The list of proteins displayed in the heatmap of Fig. 3D is available in Supplemental Table 6.

### For top 15 HDL-associated proteins, the abundance inversely associates with variability

**Fig. 4:**
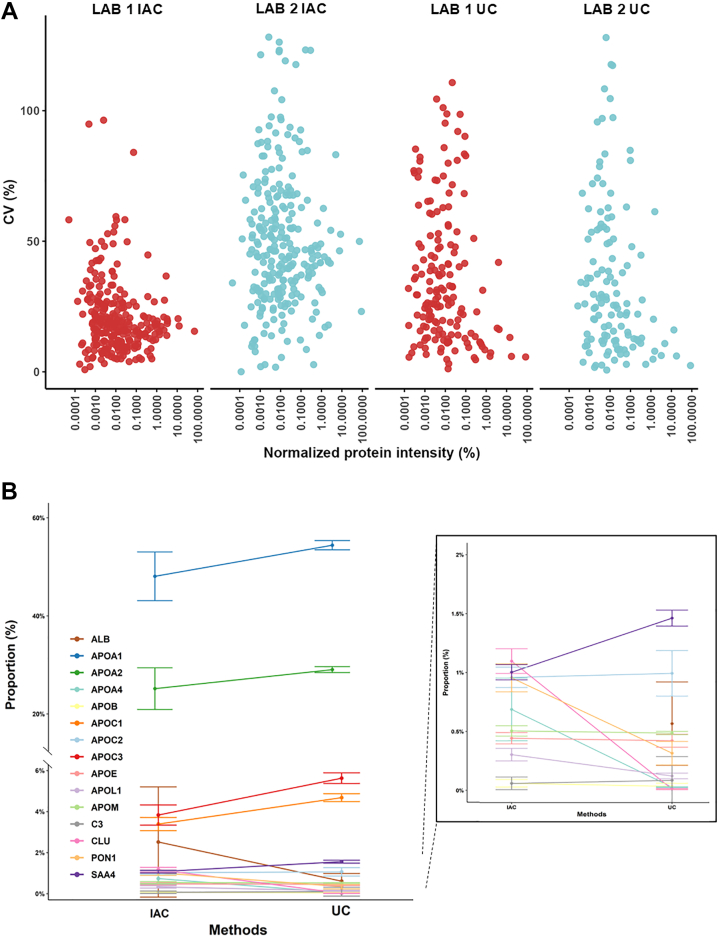
Variation and proportion of HDL proteins isolated by UC and IAC. A: Coefficient of variation (CV) of HDL proteins as a function of their abundance. The y-axis shows the CV for each protein, calculated by manual inspection of peptide areas in Skyline, whereas the x-axis represents the mean protein intensity normalized to the total HDL intensity, as provided by DIA-NN. For visualization purposes, x-axis values were log_10_-transformed. B: Average proportion of the 15 most abundant proteins across replicates from three non-consecutive experiments using IAC and UC performed in both laboratories.

Although 302 proteins were detected in this study, the vast majority of them displays low relative abundance in HDL particles. We used DIA-NN normalized intensity to calculate the relative abundance of HDL proteins within each sample. Thus, the top 15 HDL proteins accounted for 91.6% and 97.9% of the total normalized protein abundance found for IAC-HDL and UC-HDL samples, respectively, letting >200 proteins in HDL account for less than 10% of total protein amount. The number of proteins detected in HDL has raised considerably in the past 10 years, due to the improvement of the sensitivity of the mass spectrometers. The high number of variable, low abundant proteins in HDL proteome, is an important but still unresolved issue in the field. HDL is isolated from blood (which contains very high abundance proteins, such as albumin, immunoglobulins and proteins from the complement system). Many of them are known to associate with lipids. Up to date, there is no method to evaluate whether a low abundant protein belongs to HDL particles or if it is a plasma contaminant. For each protein, we used the software Skyline to verify peptide quantification (Supplemental Table 3). Thus, Fig. 4A shows the relationship between relative protein abundance and the coefficient of variation (CV), calculated after manual inspection, indicating that more abundant proteins in HDL generally exhibit lower CV. Interestingly, this trend is not consistent, especially for some low-abundant proteins, which may display low variability across isolation methods and laboratories (see below). However, the great majority of the least abundant proteins presented CV > 25%. This lack of reproducibility prevents drawing reliable conclusions about their association with HDL particles. Three main factors may contribute to this variability: (i) some low-abundance proteins may represent serum contaminants rather than true HDL components; (ii) others might belong to specific HDL subpopulations, making their isolation inherently variable; (iii) certain methods are more efficient in isolating proteins associated with HDL particles or co-eluting serum contaminants. As we cannot, at this time, determine if low-abundance protein truly belongs to HDL particles, and as this study aims to determine the variation in HDL particles within and across labs, we have decided to arbitrarily evaluate repeatability and reproducibility of the top 15 HDL proteins. Figure 4B shows the proportions of the top 15 HDL-associated proteins (the percentages are available in Supplemental Table 7, with the top 15 HDL-associated proteins in red**)**, as estimated after HDL isolation by UC and IAC. These proteins were selected based on their relative contribution to the total intensity signal in each method. APOA1 presents the highest proportion, independently of the laboratory or isolation method, representing about 54% in UC-HDL and 48% in IAC-HDL. For APOA2, the second most abundant protein in HDL particles, those abundances are around 29% for UC-HDL and 25% for IAC-HDL. The lower proportion of APOA1 and APOA2 in IAC-HDL matches the higher proportion of low-abundance proteins, such as albumin (0.6% and 2.5% of total MS signal for UC-HDL and IAC-HDL, respectively).

### Evaluation of interlaboratory variability in HDL isolation

**Fig. 5:**
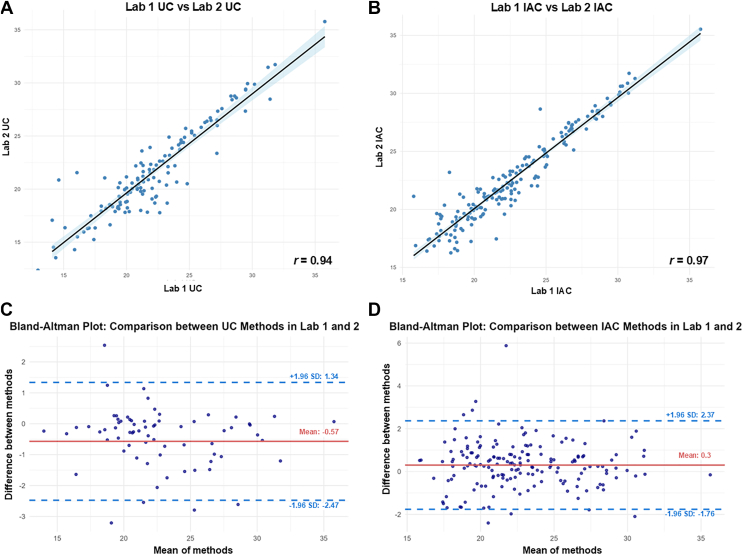
Interlaboratory agreement for the proteome of UC and IAC-isolated HDL particles. A-B: Correlation plots of log_2_-transformed intensities of all proteins identified in a representative replicate of HDL isolated by each laboratory using UC (A) or IAC (B). The Pearson correlation coefficient (*r*) is displayed. C-D: Bland-Altman plots of log_2_-transformed intensities of proteins identified on both laboratories for HDL particles isolated using either UC (C) or IAC (D). The red line is the average of all the differences between the two measurements, and the blue dashed lines represent the 95% limits of agreement.

The data obtained so far point to a dissimilar composition of HDL proteome when comparing IAC and UC isolation methodologies. Another key unresolved question regarding HDL proteome is the consistency obtained during the isolation procedure, considering intra- and interlaboratory variances. There is no study systematically evaluating figures of merit in HDL isolation procedures across different laboratories. To fill this gap, we next analyzed the distribution and the agreement for the proteins commonly obtained by Lab 1 and 2 using Pearson correlations and Bland-Altman plots (Fig. 5). For these analyses, we used the Software Skyline to manually integrate and filter peptides corresponding to proteins commonly identified in HDL particles isolated by both laboratories (a complete list of peptides quantified for each protein is available in Supplemental Table 2). This manual inspection aimed to evaluate the most reliable surrogate peptides for protein abundance, as it would be performed in clinical studies (quantification data for the proteins is available in Supplemental Table 3, with top 15 HDL-associated proteins in red). The selection of peptides ensured accurate peak boundaries and transition integration, filtering out peptides that showed poor signal quality, interference or inconsistent detection across replicates.

Pearson correlation analysis of a representative sample for UC (Fig. 5A) or IAC-isolated HDL (Fig. 5B) reveals that data obtained by the two laboratories are highly correlated, regardless of the isolation method (*r* = 0.94 and *r* = 0.97 for UC and IAC methods, respectively). Correlations obtained with the other replicates have a similar profile (Supplemental Fig. S2), showing consistency between the results obtained by the two laboratories. Agreement was also evaluated by Bland-Altman plots. Figure 5C presents the Bland-Altman plots comparing the results obtained by isolation of HDL using ultracentrifugation. The mean difference of −0.57 between the log_2_-normalized intensities of proteins indicates a small systematic bias, with laboratory 2 reporting slightly higher intensity values. The limits of agreement ranged from −2.47 to 1.34, and the majority of protein measurements fall within these limits (only 5 proteins are outside), showing agreement in the values obtained by the two laboratories for UC-isolated HDL. The Bland-Altman plots for the IAC-isolated HDL (Fig. 5D) indicates a mean difference of 0.3, with limits of agreement between −1.76 to 2.37, showing slightly higher protein abundance values in IAC-HDL isolated by Lab 1. The clustering of most measurements within the limits of agreement in HDL proteome quantification indicates results obtained by Lab 1 and Lab 2 are in agreement also for IAC-isolation methodology. Taken together, the results show that HDL isolation performed in two independent laboratories show consistent results, regardless the methods employed.

### Within-laboratory evaluation of methods agreement for HDL isolation

**Fig. 6:**
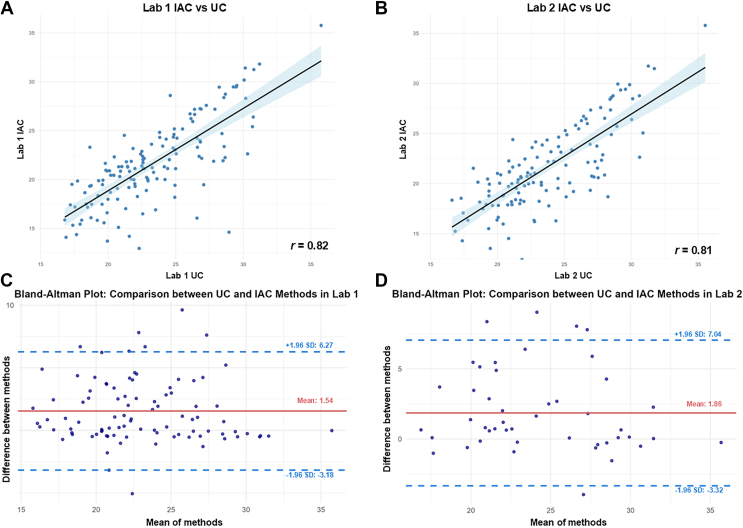
Within-laboratory agreement between IAC and UC-isolated HDL proteins. A-B: Correlation plots of log_2_-transformed intensities of all proteins identified in a representative replicate of HDL isolated by UC and IAC in laboratory 1 (A) and in laboratory 2 (B). The Pearson correlation coefficient (r) is displayed. C-D: Bland-Altman plots of log_2_-transformed intensities of proteins identified on HDL particles isolated by UC and IAC on laboratory 1 (C) and laboratory 2 (D). The red line is the average of all the differences between the two measurements, and the blue dashed lines represent the 95% limits of agreement.

We next used Pearson correlation analyses to compare the results obtained by the same laboratory using either UC or IAC methodologies for HDL isolation. Figure 6 shows the results of a representative sample. Thus, when comparing the results obtained by UC-isolated HDL with those obtained by IAC purification performed in the same laboratory, Pearson correlation coefficients of *r* = 0.81 and *r* = 0.82 were respectively obtained for Lab 1 and 2 (Fig. 6A, B). Only commonly detected proteins in HDL isolated by UC and IA were included in these analyses (n = 184). These coefficients are lower than those obtained when comparing the same methodology across distinct laboratories (*r* = 0.94 for UC and *r* = 0.97 for IAC). We also compared the level of agreement between all replicates of the two distinct methods performed in the same laboratory. Thus, the Bland-Altman plots obtained comparing isolation methods performed in Lab 1 (Fig. 6C) revealed a mean difference of 1.54 (95% limits of agreement from −3.18 to 6.34), indicating a more substantial systematic bias, accompanied by a greater variability. Similar results were obtained for the Lab 2 comparison (Fig. 6D), with a mean difference of 1.86, and limits of agreement ranging from −3.32 to 7.04. The wide agreement intervals obtained by both laboratories confirm the results obtained with correlation analysis, reinforcing that the isolation methodology is a more important determinant of the abundance of proteins in HDL than the laboratory HDL was isolated.

**Fig. 7:**
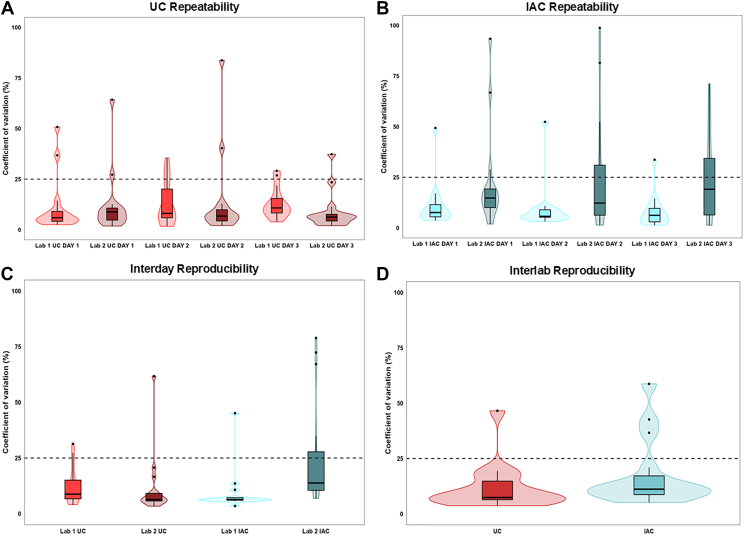
Repeatability and reproducibility of the isolation protocols performed in different laboratories. A-B: Boxplots showing the distribution of coefficients of variation (CVs) for log_2_-transformed normalized protein intensities for the 15 most abundant proteins in UC-HDL (A) and IAC-HDL (B) samples. Each boxplot represents the distribution of the CVs of three technical replicates for each of the top 15 proteins. C: CV distribution considering all nine replicates (three replicates in three non-consecutive days) for each isolation method performed in a single laboratory. D: CV distribution for all 18 replicates (nine from each laboratory), combining the results obtained by both laboratories to assess interlaboratory reproducibility. The dashed lines indicate the 25% CV threshold used as a reference for acceptable variability.

We next evaluated the repeatability of the isolation methods (Supplemental Table 3). For UC-isolated HDL samples, the daily CVs distribution in Fig. 7A indicates the majority of the top 15 HDL proteins were quantified with CVs lower than 25%, which is in accordance with the best practices for mass spectrometry quantification (20). The results obtained by Lab 1 when isolating HDL by IAC show a similar trend, with most of top 15 proteins isolated with CV<25% (Fig. 7B). However, the CVs distribution for IAC HDL isolated by Lab 2 shows a drop in repeatability as the day of isolation increase, where only 60% (9/15) of the total proteins evaluated in day 3 presented a CV lower than 25% (Fig. 7B). This result is also illustrated in the gels in Supplemental Fig. S1 and emphasizes that the control of non-specific binding to the carrier beads as well as to the antibody itself is critical. We also analyzed the intraday CVs of HDL proteins commonly detected by UC and IAC (n = 184). In UC-isolated HDL (Supplemental Fig. S3A), four of the 6 daily measurements showed at least half of the detected proteins with CVs < 25%. Overall intraday repeatability of IAC-HDL (Supplemental Fig. S3B) reflects the results obtained for the top 15 proteins (Fig. 7B). Thus, proteins isolated by IAC in Lab 1 showed greater repeatability than those isolated by Lab 2.

For each method, we also addressed the reproducibility of the 15 most abundant HDL proteins (Supplemental Table 3). Thus, Fig. 7C displays the distribution of CV values for all replicates collected across the three independent experimental days. The immunoaffinity-based isolation protocol performed in Lab 2 exhibited lower reproducibility when compared to the other measurements, with one-third of the top 15 identified proteins showing CVs above 25%. Examples of proteins with CV > 25% are albumin (ALB, 72.3%), apolipoprotein A4 (APOA4, 78.8%), apolipoprotein L1 (APOL1, 34.8%) and complement component 3 (C3, 67%). Finally, a joint analysis combining interlaboratory reproducibility of the top 15 HDL proteins on multiple days was undertaken (Fig. 7D). The results show 11 and 10 proteins with CV < 25% for UC and IAC, respectively. Thus, APOA1 (9.3% CV), APOA2 (21.5% CV), apolipoprotein E (APOE, 14.8%), apolipoprotein C1 (APOC1, 15.8%), apolipoprotein C2 (APOC2, 10.4%), apolipoprotein C3 (APOC3, 12.9%), apolipoprotein M (APOM, 11.4%), clusterin (CLU, 15.9%), paraoxonase 1 (PON1, 14.3%) and serum amyloid A4 (SAA4, 4.7%) all show CVs well below 25% for IAC. For UC, APOA1 (4.06% CV), APOA2 (9% CV), APOE (13.9% CV), apolipoprotein A4 (APOA4, 12.3%), APOC1 (12.1% CV), APOC2 (8.7% CV), APOC3 (8.3% CV), APOL1 (19.6% CV), APOM (10.8% CV), complement component 3 (C3, 7.5%), and SAA4 (7.7% CV) also show CVs below 25% (Supplemental Table 3).

Overall, our analyses show that the repeatability and reproducibility of HDL proteome measurements are strongly influenced by the isolation method and its execution. UC isolation consistently yielded more stable results across both laboratories and isolation days, particularly for the most abundant HDL-associated proteins. IAC isolation, while capable of producing reproducible results in optimized conditions, showed greater variability comparing to UC. Importantly, while protein abundance is generally inversely correlated with variability, this relationship is not uniform. Thus, from the 118 proteins with abundance below 0.1%, 40 displayed CVs below 25% for UC. For IAC, 176 protein presented abundance below 0.1%, with 37 displaying CV below 25%. Several low-abundance proteins with established roles in HDL biology, such as lecithin-cholesterol acyltransferase (LCAT), phospholipid transfer protein (PLTP), and paraoxonase 3 (PON3), were reproducibly quantified across almost every method and laboratory with CVs below 25% under specific conditions. For IAC, Lab 1: LCAT (4.6%), PLTP (3.9%), PON3 (4.1%); Lab 2: PLTP (9.7%), PON3 (12%). For UC, Lab 1: LCAT (17.7%), PLTP (11.8%), PON3 (13.2%); Lab 2: LCAT (21.8%), PLTP (10.7%), PON3 (7.2%). However, when considering interlaboratory reproducibility, only PON3 maintained CVs below 25% for both methods (12% for UC and 11.2% for IAC), whereas LCAT showed 31.7% and 45.7% for UC and IAC, respectively, and PLTP 17.7% and 27.8%. These results are summarized in Supplemental Table 8. Thus, the reproducibility is not guaranteed for all low-abundance HDL proteins and varies with both the isolation method and laboratory, emphasizing the importance of careful validation for these low abundant, but biologically relevant targets.

## Discussion

The limited consistency in the identification of HDL-associated proteins across different studies, even when the same isolation technique is used, underscores the ongoing difficulty in establishing a definitive HDL proteome, especially considering the complex dynamism of HDL particles. This inconsistency can be in part attributed to methodological differences in how HDL is isolated, which may vary substantially depending on the protocol used and the laboratory performing the procedure. Such variability affects both the yield and purity of the isolated HDL particles and its subclasses, leading to divergent proteomic profiles. In this work, by using two distinct isolation methodologies and performing a systematic interlaboratory evaluation of HDL proteome composition and isolation variability, we have shown that the major determinant of HDL proteome composition is the method of isolation.

Thus, our study showed UC- and IAC-isolated HDL proteome separate into two distinct clusters independently of laboratory or day of isolation, with almost 60% of the variance in the data explained by the method of isolation (Fig. 3C). Hierarchical clustering further confirmed this pattern, grouping samples by isolation method rather than by laboratory (Fig. 3D). Consistent with these results, size-exclusion chromatography revealed an early-eluting peak exclusively in IAC-HDL samples, regardless of the laboratory or experimental day, confirming previous results that showed the isolation of distinct HDL particles by IAC and UC (9). In addition, only 61% of all detected proteins were shared between the two isolation methods (184/302), with IAC presenting more exclusive proteins (99 vs. 19 unique to UC isolation). Notably, following our identification criteria, CETP was detected only in IAC-isolated HDL samples, suggesting that this method may capture a broader, possibly less tightly associated, protein subset. UC-isolated HDL was enriched for canonical apolipoproteins including APOA1, APOC1, APOC2, APOC3, and APOM, while IAC-processed HDL displayed higher abundance of CLU, immunoglobulins (IGHM, IGHA), and complement proteins (C3 and C4). Four main factors account for these striking differences. First, the nature of the two isolation methods is rather distinct. Shear forces and high ionic strength may strip loose proteins from UC-isolated HDL (21, 22), and lipid-binding proteins from plasma, such as ALB and SERPINA1 may have similar density (23). On the other hand, antibody-based methods may suffer from a lack of specificity, antibodies may fail to capture certain protein conformations, and contaminant proteins may non-specifically bind either the antibody or its immobilization matrix (3). Second, HDL particles are highly heterogeneous, although they were originally defined by its flotation at a density range of 1.063–1.21 g/ml (4), and later on, by its apolipoprotein composition, with two major classes of HDL particles – those containing only APOA1 (LpAI), and those containing APOA1 and APOA2 (LpA-I/LpA-II) (24, 25). A recent work showed LpAI particles can be further fractionated in large and small particles, with large particles having more proteome diversity (26). Third, since the publication of early studies concerning HDL, it became evident that HDL is composed of distinct subpopulations of particles that might be selectively favored by one or another isolation method. Indeed, using immunoaffinity isolation techniques, 16 compositional HDL subspecies with unique proteomes were isolated (7). Isolation first using nondenaturing polyacrylamide gel electrophoresis, and then immunoaffinity for APOA1 yielded 5 different HDL subspecies, and the same number was obtained using charge, size and APOA1 content to categorize HDL particles (27). Separating HDL from plasma by size using gel filtration followed by incubation with a phospholipid binding resin yielded 10 different species in HDL size range (28). Fourth, each isolation method also co-isolates a distinct set of contaminants, and it is imperative to define which proteins truly belong to HDL particles to promote advances in the field.

Importantly, the top 15 HDL proteins account for > 90% of the protein mass in HDL, regardless of the isolation method, and the variability in protein quantification is inversely associated with protein abundance. Thus, extreme caution must be taken to evaluate a minor, putative protein component of HDL in a given disease. Indeed, throughout the years, HDL proteome has been associated with many pathophysiological conditions, but validation across different studies and laboratories of such associations is still lacking. This observation highlights the challenges inherent to quantifying low-abundance HDL-associated proteins and underscores the need for careful interpretation of such data. The use of targeted proteomic methodologies may improve the reproducibility in the quantification of low-abundant HDL-associated proteins (29). Two vital steps are necessary to accomplish HDL proteome validation with disease correlation. First, the presence of a given protein biomarker in HDL must be validated (it is vital to separate HDL proteins from contaminants). Second, a quantitative, validated method for HDL proteome quantification must be employed, and the technical variability must be known (for HDL isolation and MS analyses) (15, 18, 29).

Previously, we and others (3, 8, 9, 30) evaluated how the isolation method impacts HDL proteome composition. Similar results were obtained when comparing the relative percentage of proteins of this work with those reported by Holzer *et al.* (9). Thus, IAC- isolated HDL had 56% of APOA1 in that work, comparing with 50% (Lab1) and 45% (Lab 2) in the current work, while APOA2 also displayed similar values (22% in IAC-isolated HDL in that work, compared to 22% (Lab1) and 28% (Lab2) in this work). UC- isolated HDL also provided similar results, with 53% of APOA1 and 25% of APOA2 in Holzer's work, comparing to 53% and 55% of APOA1, respectively for Labs 1% and 2%, and 25% for APOA2 obtained by both labs. However, so far, no study has addressed the composition, the abundance of proteins and the variability connected to each isolation technique. Moreover, no systematic interlaboratory evaluation has been undertaken. In this work, we have shown that isolation of HDL particles by UC is reproducible, and for the top 15 proteins, display CVs well below 25%. This CV is in accordance with best practices in proteomics for clinical samples (31, 32, 33) and it was accomplished for intra- and interday isolations, and for experiments carried out in 2 distinct laboratories. IAC-isolated HDL particles can yield an equally reproducible proteome, but the higher CVs obtained in one day of isolation in Lab 2 highlights the importance of a careful quality control process to avoid unwanted technical variance in clinical studies.

Strengths of this work include our comparative assessment of HDL proteome obtained from the two common isolation methods performed in different laboratories, and a careful evaluation of the data to establish the variability across and within the isolation methodologies. Our study also has limitations. Our work did not assess other available methods to isolate HDL, such as dextran precipitation or a combination of isolation methodologies. Also, quantifications are relative, not absolute. Moreover, we did not investigate the impact of alternative protocols widely used in the field, such as UC by two-step sequential centrifugation or IAC performed with different anti-APOA1 antibodies or bead chemistries, which may introduce additional variability. A comprehensive evaluation of these sources of variation lies beyond the scope of the present work, but represents a critical next step for translational applicability.

In summary, our findings demonstrate that, while both UC and IAC isolation methods can achieve acceptable repeatability and reproducibility, they capture distinct HDL particle populations with different proteomic profiles. The methodological impact on both protein identification and quantification emphasize that UC and IAC are not interchangeable and that careful consideration must be given to method selection based on study goals. Finally, for the first time, interlaboratory reproducibility in HDL particle isolation for proteomic analysis was reported. The interlaboratory agreement obtained for each method suggests that standardized protocols and rigorous quality control can minimize analyst-induced variability. Nonetheless, the data presented here reinforce the urgent need for consensus guidelines and standardized reference materials in HDL proteomics, to ensure comparability and reproducibility across studies and laboratories.

## Data Availability

The mass spectrometry proteomics data have been deposited to the ProteomeXchange Consortium via the PRIDE partner repository with the dataset identifier PXD066558 (34).

## Supplemental Data

This article contains supplemental data.

## Conflict of Interest

The authors declare that they do not have any conflicts of interest with the content of this article.
