# Supplementary material for: Proteomic analysis of HDL isolates reveals method-driven variability: An interlaboratory approach
Source: J Lipid Res. 2025 Dec 5;67(1):100957. doi: 10.1016/j.jlr.2025.100957 (PMC12803808; doi:10.1016/j.jlr.2025.100957)
Supplement: Supplementary Information [file mmc1.pdf]

Proteomic analysis of HDL isolates reveals method-driven variability: an interlaboratory approach

Francielle Aguiar Gomes[1&,] Douglas Ricardo Souza Junior[1&], Michael Holzer[2,3] and Graziella Eliza Ronsein[1*]

Supplementary Information

[1]Department of Biochemistry, Institute of Chemistry, University of São Paulo, São Paulo, Brazil

[2]Division of Pharmacology, Otto-Loewi Research Centre, Medical University of Graz, Graz, Austria

[3]BioTechMed Graz, Graz, Austria.

[&]These authors contributed equally to this work.

*Corresponding author

Graziella Eliza Ronsein, PhD

Department of Biochemistry, Institute of Chemistry, University of São Paulo, São Paulo, Brazil

Av. Prof. Lineu Prestes, 748, São Paulo, SP, 05508-000, Brazil

Contact phone: +55(11) 3091-2039

E-mail: ronsein@iq.usp.br

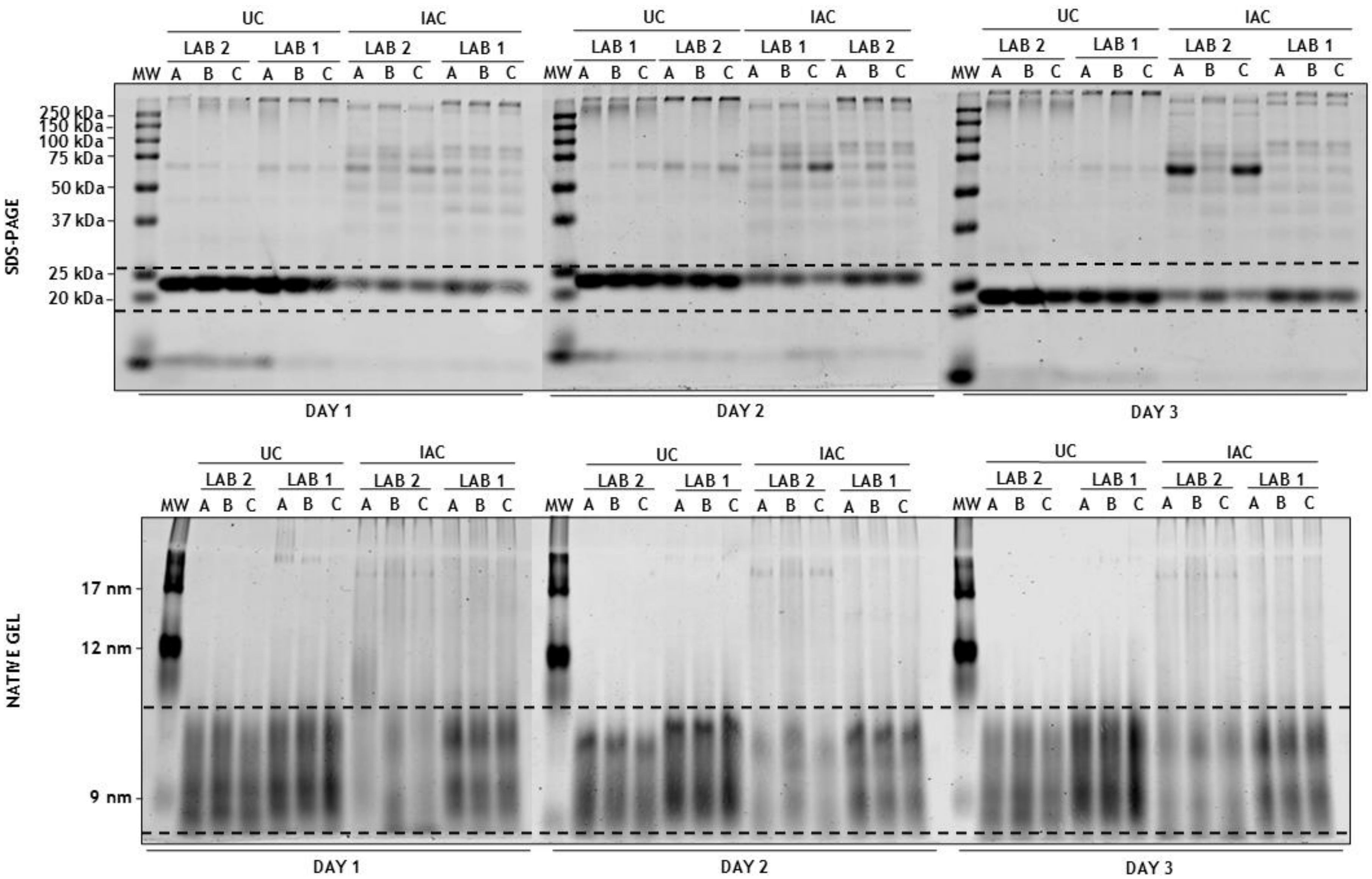

Supplementary Figure 1. Gel electrophoresis of isolated HDL by ultracentrifugation (UC) and immunoaffinity chromatography (IAC) protocols. HDL was isolated in non-consecutive days by the indicated methods from the same serum pool in both labs. The isolated HDL (5 µg protein per lane) was separated by SDS-PAGE (top panel) and native gel electrophoresis (bottom panel). The gels were stained with Commassie Blue to visualize the protein load and size distribution. Dashed lines indicate boundaries for APOA1 and HDL species, respecitvely in SDS-PAGE and native gels. Molecular weight marker (MW).

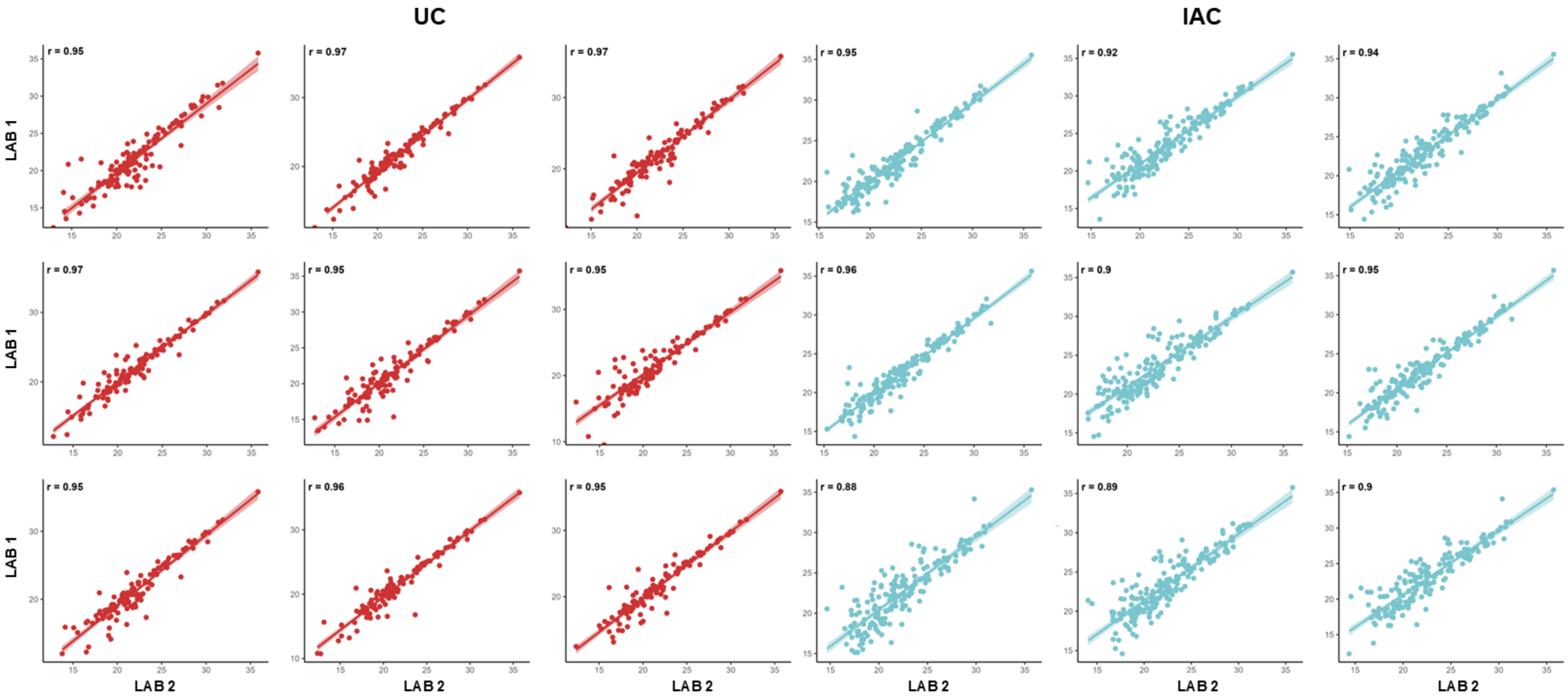

Supplementary Figure 2. Correlation between the isolation methods. Scatter plots showing the relationship between log_2-transformed protein intensities for all replicates obtained in Laboratory 1 and Laboratory 2 by UC-HDL (red) and IAC-HDL (blue). Each point represents one protein identified by both isolation methods (n = 184). Pearson correlation coefficients (r) are indicated for each comparison.

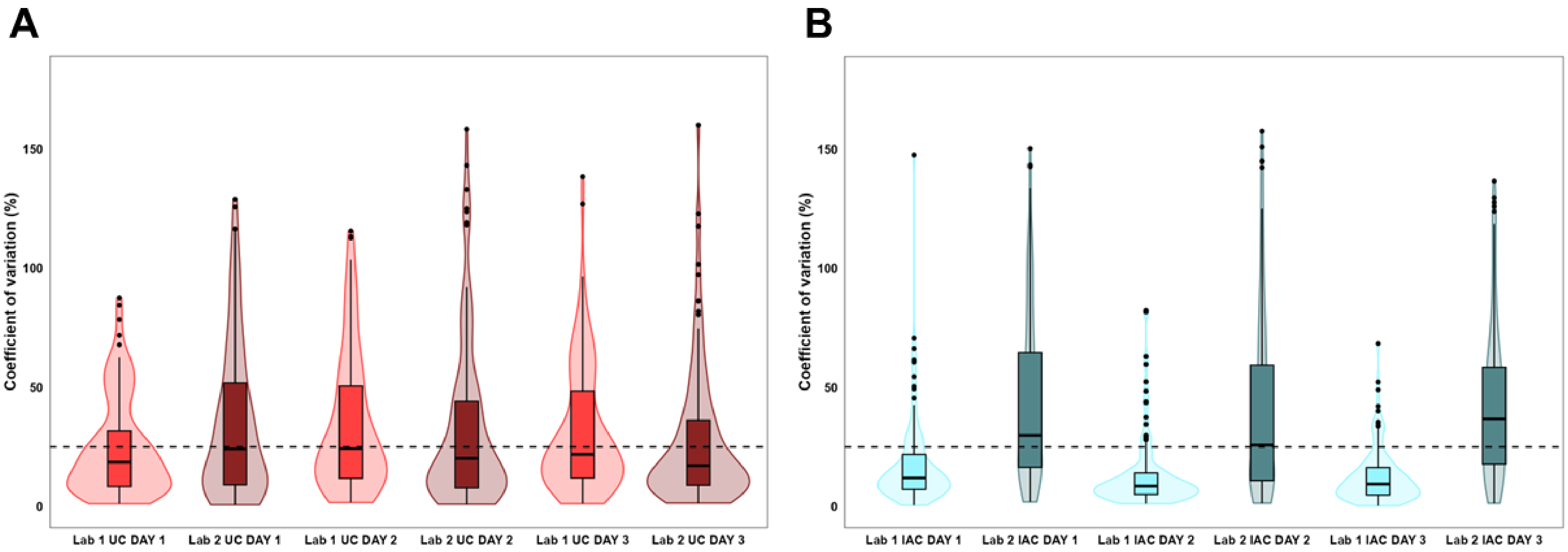

Supplementary Figure 3. Reproducibility of the isolation protocols performed in different laboratories. Distribution of coefficients of variation (CV) for the comparison of all log_2-normalized protein intensities (n = 3 replicates per day, in three independent days) for UC-HDL samples **(A),** IAC-HDL samples **(B)**. Dashed lines indicate CV of 25%.
